# Biomechanical comparison of multilevel lateral interbody fusion with and without supplementary instrumentation: a three-dimensional finite element study

**DOI:** 10.1186/s12891-017-1387-6

**Published:** 2017-02-02

**Authors:** Xilin Liu, Jun Ma, Paul Park, Xiaodong Huang, Ning Xie, Xiaojian Ye

**Affiliations:** 1 Department of Orthopedics, Changzheng Hospital, Second Military Medical University, 415 Fengyang Road, Shanghai, 200003 China; 2 Department of Neurosurgery, University of Michigan, 1500 E Medical Center Dr, Ann Arbor, MI 48109 USA

**Keywords:** Finite element analysis, Minimally invasive lateral lumbar interbody fusion, LLIF, Stand-alone, Range of motion, Stress distribution

## Abstract

**Background:**

Lateral lumbar interbody fusion (LLIF) is a popular, minimally invasive technique that is used to address challenging multilevel degenerative spinal diseases. It remains controversial whether supplemental instrumentation should be added for multilevel LLIF. In this study, we compared the kinematic stability afforded by stand-alone lateral cages with those supplemented by bilateral pedicle screws and rods (PSR), unilateral PSR, or lateral plate (LP) fixation using a finite-element (FE) model of a multi-level LLIF construct with simulated osteoporosis. Additionally, to evaluate the prospect of cage subsidence, the stress change characteristics were surveyed at cage-endplate interfaces.

**Methods:**

A nonlinear 3-dimensional FE model of the lumbar spine (L2 to sacrum) was used. After validation, four patterns of instrumented 3-level LLIF (L2-L5) were constructed for this analysis: (a) 3 stand-alone lateral cages (SLC), (b) 3 lateral cages with lateral plate and two screws (parallel to endplate) fixated separately (LPC), (c) 3 lateral cages with bilateral pedicle screw and rod fixation (LC + BPSR), and (d) 3 lateral cages with unilateral pedicle and rod fixation (LC + UPSR). The segmental and overall range of motion (ROM) of each implanted condition were investigated and compared with the intact model. The peak von Mises stresses upon each (superior) endplate and the stress distribution were used for analysis.

**Results:**

BPSR provided the maximum reduction of ROM among the configurations at every plane of motion (66.7–90.9% of intact spine). UPSR also provided significant segmental ROM reduction (45.0–88.3%). SLC provided a minimal restriction of ROM (10.0–75.1%), and LPC was found to be less stable than both posterior fixation (23.9–86.2%) constructs. The construct with stand-alone lateral cages generated greater endplate stresses than did any of the other multilevel LLIF models. For the L3, L4 and L5 endplates, peak endplate stresses caused by the SLC construct exceeded the BPSR group by 52.7, 63.8, and 54.2% in flexion, 22.3, 40.1, and 31.4% in extension, 170.2, 175.1, and 134.0% in lateral bending, and 90.7, 45.5, and 30.0% in axial rotation, respectively. The stresses tended to be more concentrated at the periphery of the endplates.

**Conclusions:**

SLC and LPC provided inadequate ROM restriction for the multilevel LLIF constructs, whereas lateral cages with BPSR or UPSR fixation provided favorable biomechanical stability. Moreover, SLC generated significantly higher endplate stress compared with supplemental instrumentation, which may have increased the risk of cage subsidence. Further biomechanical and clinical studies are required to validate our FEA findings.

**Electronic supplementary material:**

The online version of this article (doi:10.1186/s12891-017-1387-6) contains supplementary material, which is available to authorized users.

## Background

Compared with conventional open surgery, minimally invasive spinal (MIS) fusion procedures have been shown to be clinically effective and have the added benefits of a decreased hospital stay, less blood loss, decreased adjacent muscle damage, and decreased infection rate [1–4]. The lateral lumbar interbody fusion (LLIF) is a minimally invasive procedure that was developed relatively recently and is an increasingly popular treatment for multilevel degenerative spinal diseases [5, 6]. The main advantages of LLIF involve the placement of a large cage without violating the posterior elements compared with MIS transforaminal interbody fusion (TLIF). Compared to ALIF, there is decreased risk of major artery or visceral injury with intact anterior annulus and ligaments [4, 7–10]. It has also been suggested that LLIF can enhance the stability of the anterior column, create substantial indirect decompression with a restored disc and foraminal height, and significantly improve the coronal and sagittal alignment in selected de novo scoliosis cases [6, 11–15].

In previous studies of biomechanics, a laterally placed cage resulted in superior segmental stability compared to ALIF and TLIF cages. Specifically, there was a significantly reduced range of motion (ROM), without the requirement for supplemental instrumentation. However, most of these studies were limited to kinematic analysis in single-level conditions [16, 17]. When used clinically in multilevel procedures, however, the stand-alone LLIF construct is likely to have limited stability without supplemental fixation and is likely to have more limited curve correction in cases with scoliosis [14, 18, 19]. Despite the reported satisfactory clinical outcomes in scoliosis cases, an elevated incidence of cage subsidence was observed in stand-alone LLIF compared to those with supplemental fixation, particularly for older patients with impaired bone mineral density (BMD) [13, 20–26]. High-grade subsidence could result in the re-stenosis of the intervertebral foramen and a loss of segmental lordosis, leading to persistent back pain or radiculopathy and a need for revision surgery [20, 27].

To prevent subsidence and pseudoarthrosis, supplementary fixation, typically by pedicle screw and rod (PSR) fixation, has been performed. The use of PSR to significantly reduce ROM has been verified [16, 28]. The disadvantages of PSR, however, include the need for a second incision with added exposure-related morbidity, an extended anesthesia time, and an increased cost for a multilevel LLIF surgery. Although lateral supplementary fixation using a plate and bi-cortical vertebral body screws is also an option, it is less rigid and may not be as effective for multilevel cases or scoliosis correction [13, 29].

Currently, there is a paucity of literature evaluating the need for supplementary instrumentation after multilevel LLIF. The objective of this study was to evaluate the biomechanical stability of stand-alone multi-level LLIF versus multi-level LLIF with several types of supplemental instrumentation and to analyze the factors associated with subsidence.

## Methods

**Fig. 1:**
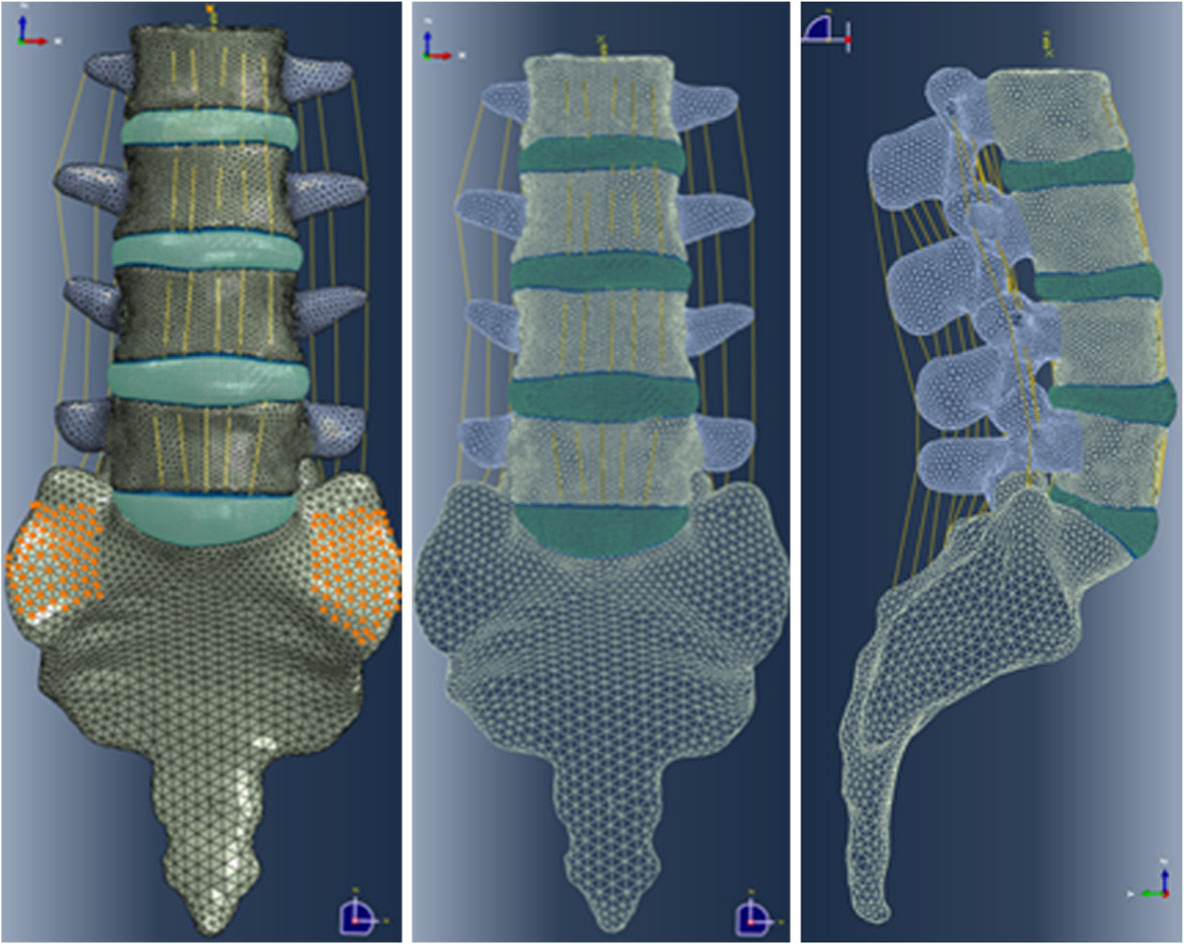
Non-linear 3-dimensional FE model of L2-sacrum vertebra

**Fig. 2:**
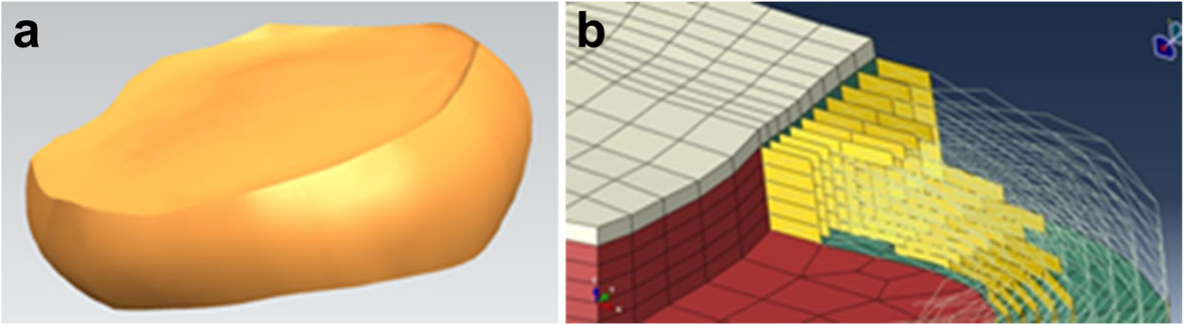
Construction of the disc model. **a** The constructed discs are composed of the nucleus pulposus (NP) and 56% annulus fibrosus (AF). **b** The collagen fibers that support the AF matrix were angled at 30 to 45° with respect to the horizontal plane and varied from the inner to outer lamina of the AF

A nonlinear 3-dimensional FE model was used for analysis. The geometry of the lumbosacral spine was reconstructed from 1-mm-thick computerized tomography (CT) scans of a healthy adult male. The CT scan images were processed with commercial software (Mimics 15.0; Materialise, Leuven, Belgium) and transformed into a solid model. After repair, denoise and spheroidality (Geomagic Studio12.0; Geomagic, SC, USA), the data were assembled (Pro/E5.0; PTC, MA, USA) into the 3D finite element model consisting of the L2-sacrum vertebra (Fig. 1). The FEM construct comprised the L2-S vertebral bodies, posterior elements (including cortical and cancellous bone), intervertebral discs, endplates, and ligamentous system (anterior longitudinal ligament, posterior longitudinal ligament, capsular ligament, intertransverse ligament, ligamentum flavam, interspinous and supraspinous ligament). The discs were defined to be composed of 44% nucleus pulposus (NP) and 56% annulus fibrosus (AF) based on histological data. The elastic behavior of the AF was simulated using a hyper-elastic Mooney-Rivlin formulation with eight annulus fiber layers modeled in a radial orientation [30]. The collagen fibers of the AF matrix were angled at 30° to 45° with respect to the horizontal plane and varied from the inner to outer lamina of the AF (Fig. 2). The nonlinear structural behavior of the spinal ligaments was modeled using the Maxwell–Kelvin–Voigt visco-elastic law. Both the annulus fibers and ligaments were set to be truss elements subjected only to tensile load. The surface-surface contact elements were used to simulate facet joints, and the coefficient of friction was set at 0.1 [31]. The amount of tetrahedral mesh is 249,049, the amount of hexahedral mesh is 46,332, the two-dimensional quadrilateral shell is 24,336, and there are 99,042 one-dimensional truss elements, summing to 418,759 elements and 109,583 nodes in total. The “osteoporotic spine” was modeled by simulating the loss of elastic modulus of normal bone, and 33 and 66% of the elastic modulus was reduced for the cortical and cancellous bone, respectively [32].

### FEM validation

The ROM data were compared to the results of a cadaveric biomechanical study conducted by Shim et al. [33], who applied a similar load in flexion, extension, lateral bending, and axial rotation. The intact FE model was confirmed to be valid because the calculated ROM was close in magnitude to what has been reported in the literature.

### FEM with implants

**Fig. 3:**
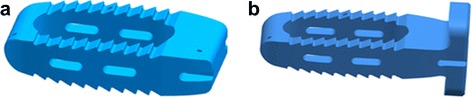
Configuration of the designed lateral cage (LC); (**a**) lateral cage with 2-hole lateral plate (LPC); (**b**) The titanium alloy screws for LPC are not shown

**Fig. 4:**
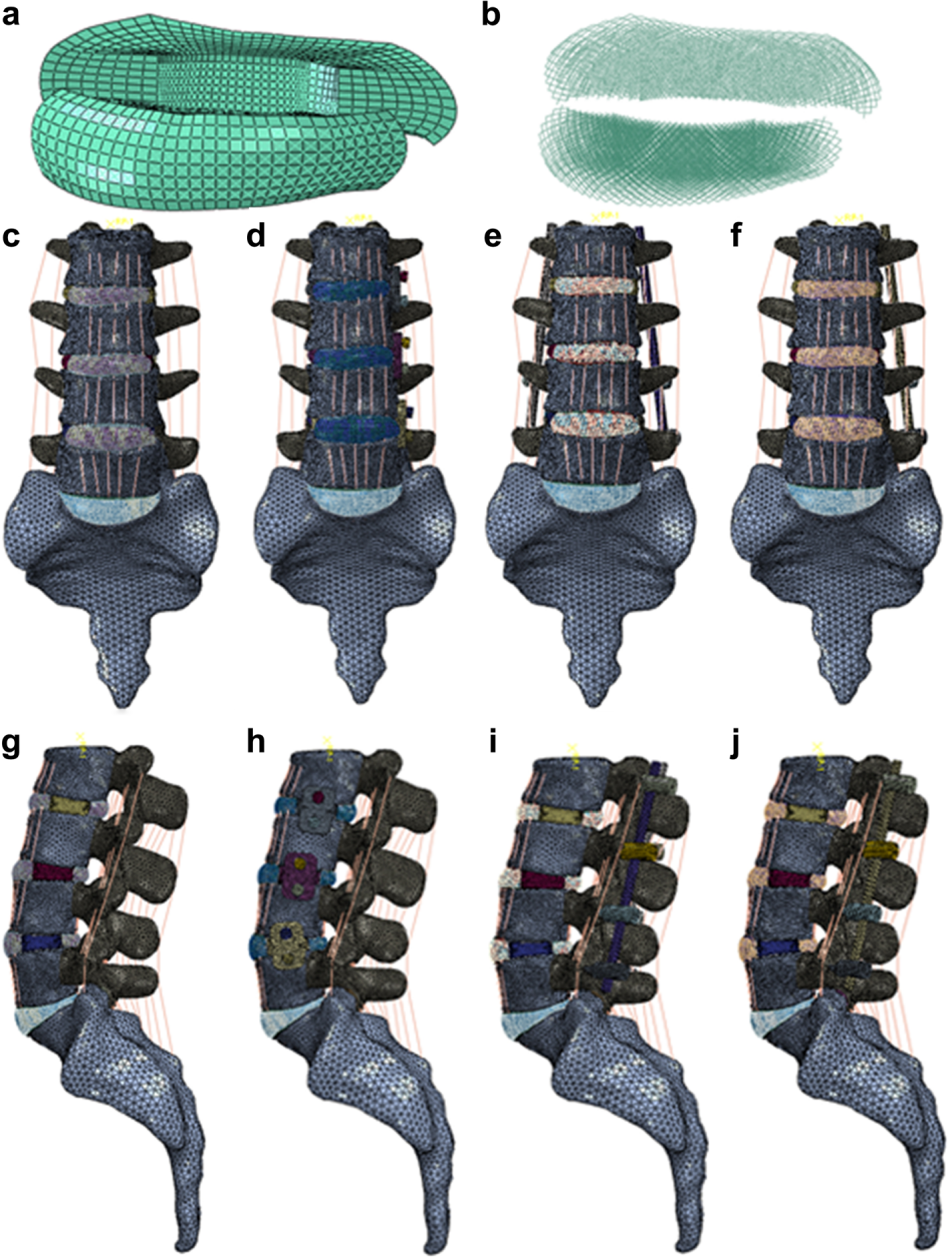
Simulation of the surgical procedure of L2-L5 LLIF. **a**, **b**: The surgical procedure was simulated as partial resection of lateral AF, removal of whole NP and cartilaginous endplate. **c**-**j**: anterior-posterior and lateral view of 4 patterns of instrumented model. **c**, **g**: 3 stand-alone lateral cages (SLC); **d**, **h**: 3 lateral cages with plate and screws(LPC); **e**, **i**: 3 lateral cages with bilateral pedicle screws and rods (LC + BPSR); **f**, **j**: 3 lateral cages with unilateral PSR system (UPSR)

A lateral cage (LC) and a lateral cage with two-hole lateral plate (LPC) were defined using commercial software (UG NX8.0, Siemens PLM Software, Germany) (Fig. 3). The material of the LC and LPC was defined as polyether ether ketone (PEEK), and the screws for LPC and the posterior pedicle screw rod system (PSR) were designated titanium alloy. The configuration of the LC and LPC were similar to commercial LLIF cages, with a width of 22 mm and a “roughened” endplate surface. The material properties of the implant components are listed in Table 1 [34]. The surgical procedure involved in the typical L2-L5 LLIF was simulated and involved resection of the lateral (left) annulus and removal of NP and cartilaginous endplate in conjunction with contralateral annulus release and the subsequent insertion of a cage (cage was placed at the mid-anterior part of disc space) with or without additional fixation. The height and lordosis of the cages were adjusted based on the preoperative height and segmental angle of the targeted discs (Fig. 4). In addition to the L2-S intact and osteoporotic spine models, the following four patterns of instrumented 3-level-constructs (L2-L5) were included in our FE study (Fig. 4): three standalone lateral cages (SLC), three lateral cages with lateral plate and two screws (parallel to endplates) fixation (LPC), three lateral cages with bilateral pedicle screw and rod fixation (LC + BPSR), and three lateral cages with unilateral pedicle screw and rod fixation (LC + UPSR). The diameter of the pedicle screws was 6.5 mm, and the lengths of the screws were set to reach the anterior cortex of the vertebral body. The UPSR was placed at the ipsilateral side of the discectomy (left). The contact between the pedicle screw and bone (pedicle and vertebral body) was set as an “embedded” coupling constraint, and a “tie” constraint was used to simulate the cage and endplate interface.

**Table 1:** Material properties of implant components

	Elastic modulus (MPa)	Poisson’s ratio ν	Cross-sectional Area (mm^2)^
Cortical bone of vertebral body	12000	0.3	/
Cancellous bone of vertebral body	100	0.2	/
Pedicle	3500	0.25	/
Facet joints	15	0.45	/
Endplate	24	0.25	/
Nuclear pulposus	1	0.499	/
Annulus fibrosus	4.2	0.45	/
Fibers of Annulus fibrosus	175	/	0.76
Anterior longitudinal ligament	7.8	/	63.7
Posterior longitudinal ligament	1	/	20
Ligamentum flavum	1.5	/	40
Capsular ligaments	7.5	/	30
Intertransverse ligaments	10	/	1.8
Interspinous ligaments	1	/	40
Supraspinous ligaments	3	/	30

### Loading and boundary conditions

The multi-level FE model from L2 through the entire sacrum spine was used for analysis. For all of the implanted and intact constructs, the contact nodes of the sacrum-pelvis-femoral head were defined to be rigidly fixed, and the loads were applied on the upper surface of the L2 endplate. An axial compressive preload of 400 N was set, and a torsional moment of 7.5 N-m was imposed to simulate the motions of flexion, extension, left bending (LB), right bending (RB), and axial rotation (AR). The loading parameters were based on a previous study [35]. After numerical calculation, the segmental and overall ROM of each implanted condition were investigated and compared with the intact model. The peak von Mises stress on each (superior) endplate and the stress distribution were also used for analysis. Because this study did not intend to evaluate the issue of long-term disc degeneration, the adjacent segmental ROM and disc stress characteristics were not included in the investigation.

## Results

### Range of motion

#### Segmental range of motion

##### L2-L3 ROM

**Fig. 5:**
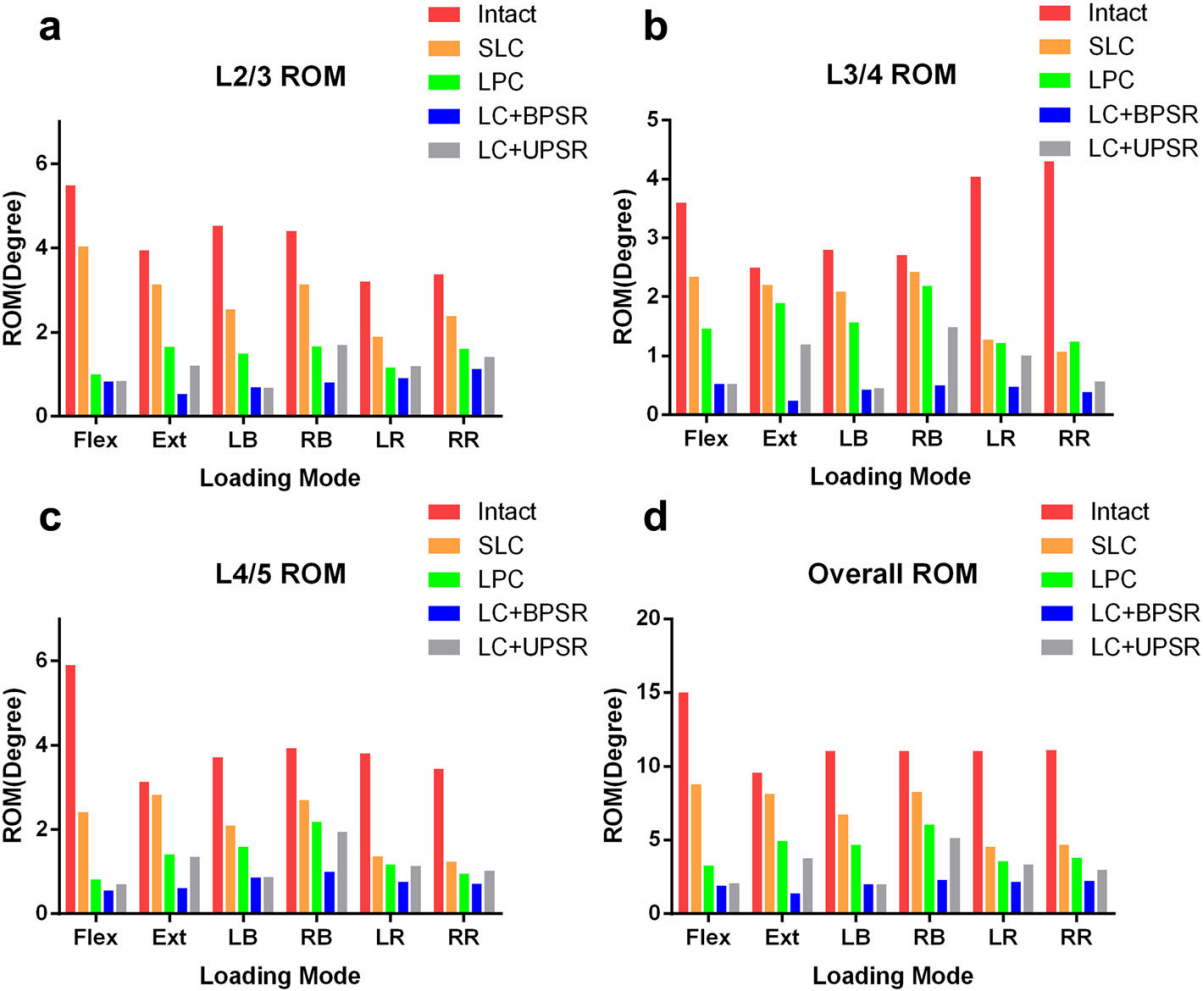
Intersegmental and overall range of motion (ROM) of LLIF constructs with SLC, LPC, LC + BPSR and LC + UPSR. **a**, L2/3 ROM; **b**, L3/4 ROM; **c**, L4/5 ROM; **d**, overall ROM. Flex = flexion; Ext = extension; LB = left bending; RB = right bending; LR = left rotation; RR = right rotation. SLC = stand-alone lateral cages (SLC); LPC = lateral cages with plate and screws; LC + BPSR = lateral cage with bilateral pedicle screws and rods; LC + UPSR = lateral cages with unilateral pedicle screw and rod fixation

At L2-L3 (Fig. 5a), all of the simulated models significantly reduced the segmental ROM compared with the intact model. However, the ROM of the standalone lateral cage (SLC) was greater than that of the latter three constructs with additional fixation (1.9–5.9 times in flexion/extension, 1.7–3.9 times in LB and 1.5–2.1 times in AR). The cage with LPC fixation was found to have greater ROM restriction in RB and LR but was generally less stable compared to posterior fixation. Even compared with the UPSR group, the LPC model increased ROM by 14.3% in RR and 208.8% in FE.

##### L3-L4 ROM

The characteristics of the L3-L4 ROM are shown in Fig. 5b. In flexion and axial rotation, the ROM of the four implanted models was significantly lower than that of the intact model, ranging from 11% (BPSR, extension) to 65% (SLC, flexion) in the intact group. Nevertheless, more limited ROM restriction was found in the LPC and SLC groups. In extension and right bending, the LPC model reached 76.1 and 80.5% of the intact ROM, respectively, and basically no significant difference in ROM was found between the SLC and intact groups (88.4 and 89.4% of intact ROM, respectively).

##### L4-L5 ROM

At L4-L5 (Fig. 5c), the constructs with additional fixation (LPC, BPSR, UPSR) all had satisfactory ROM restrictions compared with the SLC and intact models. Among these, the BPSR group provided the largest reduction of ROM, by 81–91% in FE, 75–77% in LB, and 80% in AR, compared with the intact model. In AR and lateral bending, the SLC afforded a similar ROM restriction to that of lateral and posterior fixation, but the same results were not found in other loading modes. Particularly in extension, there was no significant difference in ROM between the SLC and intact models (2.82° and 3.13°, respectively).

### Overall range of motion

The overall ROM (Fig. 5d) of the L2-L5 construct was obtained by the integration of the intersegmental data. Similar results were found compared to the segmental ROM testing, in which the SLC provided only 14.8 and 25.3% of overall ROM reduction in extension and LB, respectively, whereas BPSR provided at least 79.2% reduction for each loading mode.

### Endplate stresses analysis

**Fig. 6:**
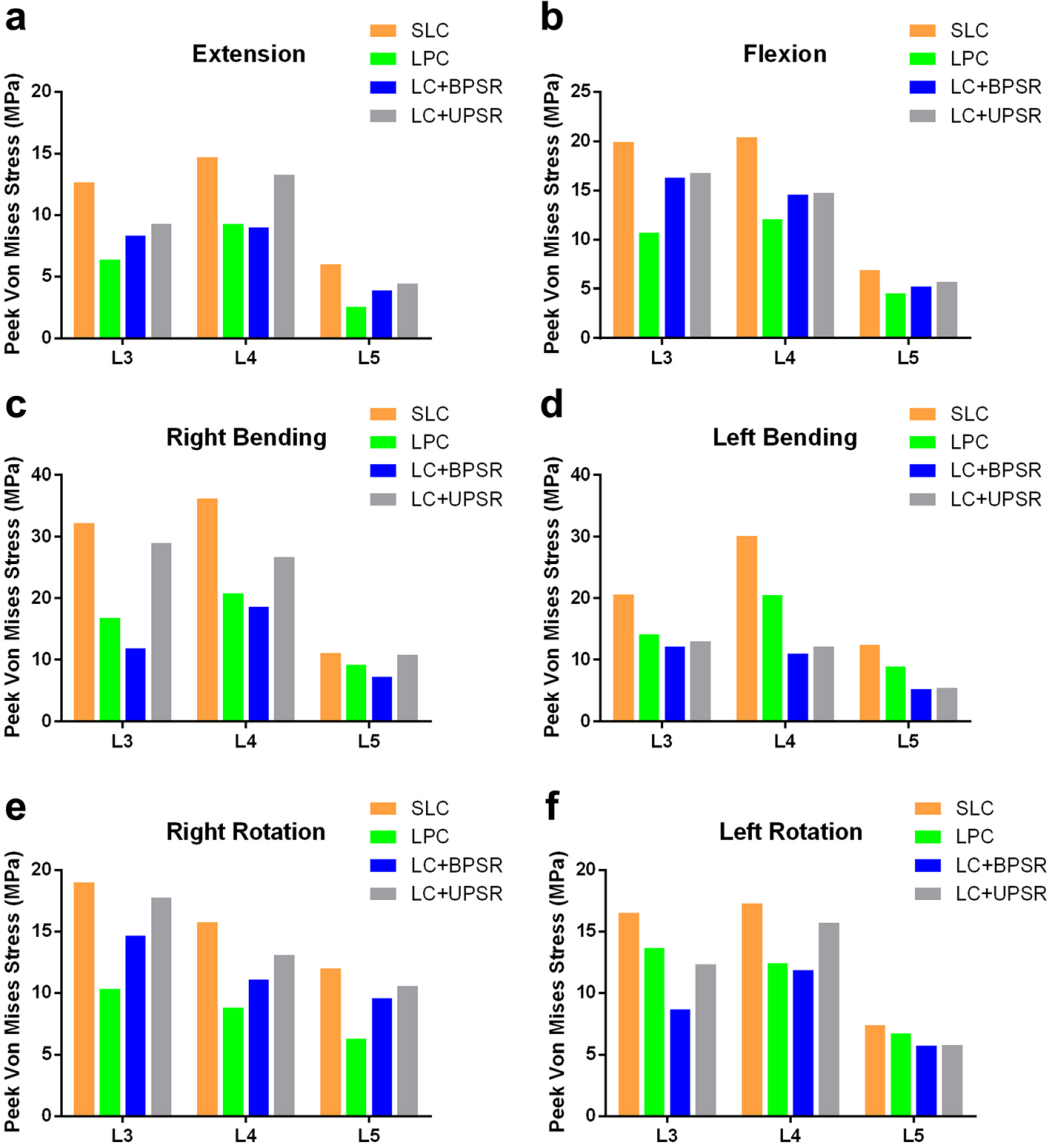
Stresses of L3, L4 and L5 superior endplates in LLIF constructs with SLC, LPC, LC + BPSR and LC + UPSR under different conditions. **a**, extension; **b**, flexion; **c**, right bending; **d**, left bending; **e**, right rotation; **f**, left rotation. SLC = stand-alone lateral cages (SLC); LPC = lateral cages with plate and screws; LC + BPSR = lateral cage with bilateral pedicle screws and rods; LC + UPSR = lateral cages with unilateral pedicle screw and rod fixation

**Fig. 7:**
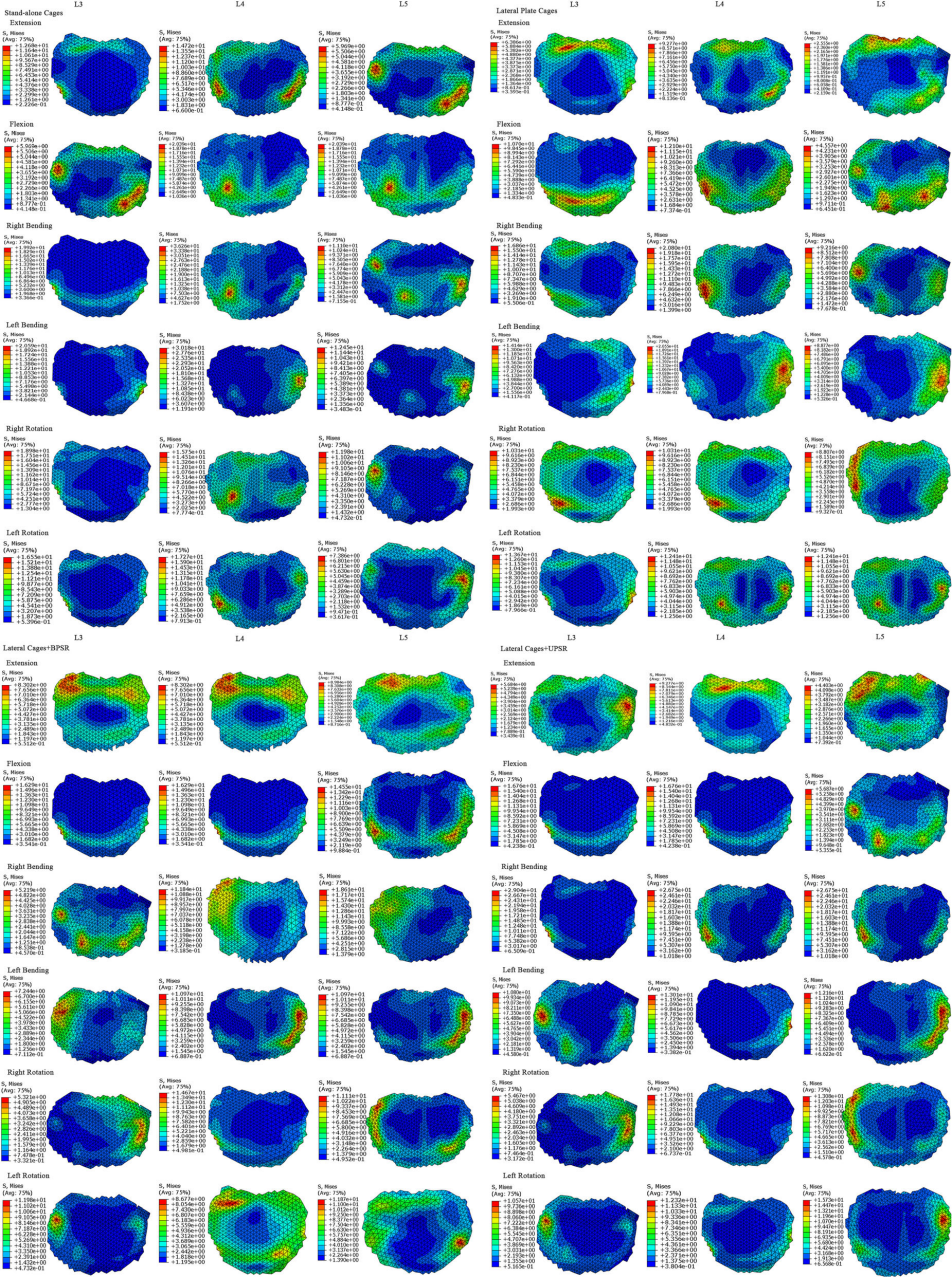
Stress distribution of L3, L4 and L5 superior endplate LLIF constructs with SLC, LPC, LC + BPSR, and LC + UPSR fixation

Because cage subsidence usually occurs inferiorly according to previous clinical reports [20, 22], only the superior endplate stress was analyzed. Data on the peak von Mises stresses of L3, L4 and L5 superior endplates are shown in Fig. 6. In all loading modes, standalone lateral cages generated greater endplate stresses than did any of the other multilevel LLIF models with supplemental fixation. For the L3, L4 and L5 endplates, the peak endplate stresses caused by the SLC construct exceeded the BPSR group by 52.7, 63.8, and 54.2% in flexion, respectively (by 22.3, 40.1, and 31.4% in extension, 170.2, 175.1, and 134.0% in lateral bending, and 90.7%, 45.5%, 30.0% in axial rotation, respectively). For lateral cages with supplementary fixation, the endplate stresses provided by unilateral pedicle fixation were slightly higher than those of LPC and BPSR model, but the difference was not significant. Figure 7 demonstrates the stress distribution of each endplate in mode of flexion, extension, lateral bending and axial rotation. In all loading modes, the stresses tended to be more concentrated at the periphery of the endplates. However, the higher stresses were located slightly more centered at the anterior lateral site of each endplate for the SLC group compared with the other three configurations, and the maximal endplate stress was noted in the SLC construct as 36.3 MPa with right bending at the L3-L4 level.

## Discussion

The minimally invasive LLLIF, although it is effective, shares common challenges with other interbody fusion techniques, including cage migration, intersegmental nonunion, and cage subsidence [27, 36–38]. Undesirable outcomes, including a higher subsidence rate and pseudoarthrosis, were more often observed in standalone-cage or multilevel LLIF cases in previous studies. Consequently, the purpose of this study was to determine the impact of supplemental fixation on biomechanical stability and subsidence in multi-level LLIF.

In a previous cadaveric study, Cappuccino et al. demonstrated that additional bilateral PSR provided the maximum reduction in ROM for the single level LLIF construct compared with the standalone cage, lateral plate, and unilateral PSR fixation [16]. Pimenta et al., however, found that the stand-alone cage provided at least a comparable reduction in ROM to a TLIF with bilateral PSR using a wider (26 mm) lateral cage [17]. Conversely, in Fogel’s L4-L5 spondylolisthesis cadaver model, the stand-alone cage reduced only approximately 23% ROM of the normal spine and significantly increased the anterior-posterior (interbody) displacement [39]. In Nayak’s study of two-level constructs, similar but slightly lower rates of ROM reduction were observed relative to Cappuccino’s findings, especially for LP fixation in lateral bending [40]. In this investigation, the stand-alone condition and levels with endplate fracture were not included for analysis.

In the present FEA study, an osteoporotic lumbar spine was modeled, which is a more realistic representation of the typically symptomatic spine and is more prone to complications such as implant failure or cage subsidence [24]. The results of this study indicate that all of the supplemental instrumented models enhanced the construct stability compared with the intact spine. However, the degree of stability was considerably different between the models. Predictably, the construct with BPSR provided the maximum reduction in ROM among all configurations at every plane of motion, ranging from 66.7 to 90.9% of the intact spine. Our data also show that the UPSR system has a favorable ability to enhance global stability, although mildly decreased ROM reduction was found in right bending, reducing 45.0% of ROM at L3/4 and 53.5% for the whole construct. In contrast, LPC fixation and stand-alone cages provided less ROM restriction than did the BPSR and UPSR constructs. A marked disparity between constructs was found in extension, where LPC reduced only 23.9% at L3/4 and 48.3% for the overall ROM, and SLC afforded less than 20% of ROM reduction compared with the intact model.

To optimize spinal fusion, instrumentation should provide biomechanical stability and also prevent endplate failure [41–43]. The results from this study demonstrate that LLIF cages generate lower endplate stress than do those reported in TLIF and ALIF FE studies [28, 44–46]. This result may be due to the theory that the lateral cage has a more favorable stress sharing mechanism because of its broader configuration. In LLIF, the endplates are more stressed at the strengthened peripheral region as the ideal lateral cage is placed across the vertebral cortical ring, which may theoretically reduce the risk of endplate failure. The clinically reported radiographic subsidence rate of LLIF is approximately 8% (14 of 178) [47] and 8.8% (21 of 238) [20] per fusion level, whereas that of TLIF cages was approximately 14.8% [36].

To our knowledge, this study is the first study to investigate both kinematic and load sharing characteristics for multilevel LLIF. The findings of the present study imply that stand-alone cages and LPC fixation may not provide adequate stability in multi-level LLIF. In addition, increased peak endplate stress was found in SLC models, which exceeded the additional instrumented models by up to 133.6, 175.1 and 90.7% in flexion/extension, lateral bending and axial rotation, respectively. Our findings are potentially supported by the recent clinical research conducted by Malharm et al., who proposed an algorithm to evaluate the need for additional instrumentation [18]. According to their study, preoperative defects such as osteoporosis, instability, spondylolisthesis and three or more fusion levels were independent indicators for posterior fixation in LLIF surgery.

The limitations of this study are typical for finite element studies. FEA cannot precisely recreate biomechanical features such as the increased loading of body weight and the influence of the paraspinal muscle. The modeling also does not accurately depict complex conditions such as collapsed disc height, spondylolisthesis, loss of lumbar lordosis or kyphosis, coronal or rotational scoliosis, strained ligaments, osteophytes, or degenerative facet joints. Finally, the in vivo vertical micro-translation of cages at the early stage of interbody fusion was simplified as a tie connection between interfaces, and the load carried by autografts within the cage was not elaborately simulated. Moreover, the model was limited to detect the instant features of static biomechanics after surgery. Because a repetitive load or material fatigue was not considered, additional FE models or biomechanical studies are required for a more long-term evaluation of LLIF.

## Conclusions

In conclusion, the results of the present study indicate that stand-alone lateral cages and supplementary lateral plate fixation provide only limited ROM restriction for the multilevel LLIF constructs, whereas BPSR or UPSR fixation provide favorable biomechanical stability. Moreover, SLC generates significantly higher endplate stress compared with supplemental instrumentation, which may cause an increase in the risk of cage subsidence. Further biomechanical and clinical studies are required to validate our FEA findings.

## Additional files

Additional file 1: Original data of ROM for each LLIF construct with different supplemental instrumentation. (XLSX 58 kb)
Additional file 2: Original data of endplate stress for each LLIF construct in different motions. (XLSX 23 kb)

## Abbreviations

AF: Annulus fibrosus
ALIF: Anterior interbody fusion
AR: Axial rotation
BMD: Bone mineral density
BPSR: Bilateral pedicle screws and rods fixation
FE: Finite-element
LB: Left bending
LC: Lateral cage
LLIF: Lateral lumbar interbody fusion
LPC: Lateral plate and cage
LR: Left rotation
MIS: Minimally invasive spinal
NP: Nucleus pulposus
PEEK: Polyether ether ketone
PSR: Pedicle screw rod
RB: Right bending
ROM: Range of motion
RR: Right rotation
SLC: Stand-alone lateral cage
TLIF: Transforaminal interbody fusion
UPSR: Unilateral pedicle screws and rod fixation

## References

[1] O’Toole JE, Eichholz KM, Fessler RG (2009). Surgical site infection rates after minimally invasive spinal surgery. J Neurosurg Spine.

[2] Park P, Okonkwo DO, Nguyen S, Mundis Jr GM, Than KD, Deviren V, La Marca F, Fu KM, Wang MY, Uribe JS, et al. Can a minimal clinically important difference be achieved in elderly patients with adult spinal deformity who undergo minimally invasive spinal surgery? World Neurosurg. 2016;86:168-72. 10.1016/j.wneu.2015.09.072 26431736

[3] Fessler RG, O’Toole JE, Eichholz KM, Perez-Cruet MJ (2006). The development of minimally invasive spine surgery. Neurosurg Clin N Am.

[4] Rodriguez-Vela J, Lobo-Escolar A, Joven E, Munoz-Marin J, Herrera A, Velilla J (2013). Clinical outcomes of minimally invasive versus open approach for one-level transforaminal lumbar interbody fusion at the 3- to 4-year follow-up. Eur Spine J.

[5] Ozgur BM, Agarwal V, Nail E, Pimenta L (2010). Two-year clinical and radiographic success of minimally invasive lateral transpsoas approach for the treatment of degenerative lumbar conditions. SAS J.

[6] Anand N, Baron EM, Khandehroo B, Kahwaty S (2013). Long-term 2- to 5-year clinical and functional outcomes of minimally invasive surgery for adult scoliosis. Spine.

[7] Quraishi NA, Konig M, Booker SJ, Shafafy M, Boszczyk BM, Grevitt MP, Mehdian H, Webb JK (2013). Access related complications in anterior lumbar surgery performed by spinal surgeons. Eur Spine J.

[8] Schizas C, Foko’o N, Matter M, Romy S, Munting E (2009). Lymphocoele: a rare and little known complication of anterior lumbar surgery. Eur Spine J.

[9] Hey HW, Hee HT (2015). Open and minimally invasive transforaminal lumbar interbody fusion: comparison of intermediate results and complications. Asian Spine J.

[10] Klingler JH, Volz F, Kruger MT, Kogias E, Rolz R, Scholz C, Sircar R, Hubbe U (2015). Accidental Durotomy in minimally invasive transforaminal lumbar interbody fusion: frequency, risk factors, and management. ScientificWorldJournal.

[11] Anand N, Baron EM, Kahwaty S (2014). Evidence basis/outcomes in minimally invasive spinal scoliosis surgery. Neurosurg Clin N Am.

[12] Baghdadi YM, Larson AN, Dekutoski MB, Cui Q, Sebastian AS, Armitage BM, Nassr A (2014). Sagittal balance and spinopelvic parameters after lateral lumbar interbody fusion for degenerative scoliosis: a case-control study. Spine.

[13] Berjano P, Lamartina C (2013). Far lateral approaches (XLIF) in adult scoliosis. Eur Spine J.

[14] Caputo AM, Michael KW, Chapman TM, Jennings JM, Hubbard EW, Isaacs RE, Brown CR (2013). Extreme lateral interbody fusion for the treatment of adult degenerative scoliosis. J Clin Neurosci.

[15] Park P, Wang MY, Lafage V, Nguyen S, Ziewacz J, Okonkwo DO, Uribe JS, Eastlack RK, Anand N, Haque R (2015). Comparison of two minimally invasive surgery strategies to treat adult spinal deformity. J Neurosurg Spine.

[16] Cappuccino A, Cornwall GB, Turner AW, Fogel GR, Duong HT, Kim KD, Brodke DS (2010). Biomechanical analysis and review of lateral lumbar fusion constructs. Spine.

[17] Pimenta L, Turner AW, Dooley ZA, Parikh RD, Peterson MD (2012). Biomechanics of lateral interbody spacers: going wider for going stiffer. ScientificWorldJournal.

[18] Malham GM, Ellis NJ, Parker RM, Blecher CM, White R, Goss B, Seex KA. Maintenance of segmental lordosis and disc height in standalone and instrumented extreme lateral interbody fusion (XLIF). Clin Spine Surg. 2016. doi: 10.1097/BSD.0b013e3182aa4c94. 10.1097/BSD.0b013e3182aa4c94 28207620

[19] Anand N, Baron EM, Khandehroo B (2014). Limitations and ceiling effects with circumferential minimally invasive correction techniques for adult scoliosis: analysis of radiological outcomes over a 7-year experience. Neurosurg Focus.

[20] Le TV, Baaj AA, Dakwar E, Burkett CJ, Murray G, Smith DA, Uribe JS (2012). Subsidence of polyetheretherketone intervertebral cages in minimally invasive lateral retroperitoneal transpsoas lumbar interbody fusion. Spine.

[21] Marchi L, Abdala N, Oliveira L, Amaral R, Coutinho E, Pimenta L (2012). Stand-alone lateral interbody fusion for the treatment of low-grade degenerative spondylolisthesis. ScientificWorldJournal.

[22] Marchi L, Abdala N, Oliveira L, Amaral R, Coutinho E, Pimenta L (2013). Radiographic and clinical evaluation of cage subsidence after stand-alone lateral interbody fusion. J Neurosurg Spine.

[23] Ahmadian A, Bach K, Bolinger B, Malham GM, Okonkwo DO, Kanter AS, Uribe JS (2015). Stand-alone minimally invasive lateral lumbar interbody fusion: multicenter clinical outcomes. J Clin Neurosci.

[24] Tempel ZJ, Gandhoke GS, Okonkwo DO, Kanter AS (2015). Impaired bone mineral density as a predictor of graft subsidence following minimally invasive transpsoas lateral lumbar interbody fusion. Eur Spine J.

[25] Castro C, Oliveira L, Amaral R, Marchi L, Pimenta L (2014). Is the lateral transpsoas approach feasible for the treatment of adult degenerative scoliosis?. Clin Orthop Relat Res.

[26] von Keudell A, Alimi M, Gebhard H, Hartl R (2015). Adult degenerative scoliosis with spinal stenosis treated with stand-alone cage via an extreme lateral transpsoas approach; a case report and literature review. Arch Bone Joint Surg.

[27] Joseph JR, Smith BW, La Marca F, Park P (2015). Comparison of complication rates of minimally invasive transforaminal lumbar interbody fusion and lateral lumbar interbody fusion: a systematic review of the literature. Neurosurg Focus.

[28] Ambati DV, Wright EK, Lehman RA, Kang DG, Wagner SC, Dmitriev AE (2015). Bilateral pedicle screw fixation provides superior biomechanical stability in transforaminal lumbar interbody fusion: a finite element study. Spine J.

[29] Tender GC (2014). Caudal vertebral body fractures following lateral interbody fusion in nonosteoporotic patients. Ochsner J.

[30] Schmidt H, Heuer F, Simon U, Kettler A, Rohlmann A, Claes L, Wilke HJ (2006). Application of a new calibration method for a three-dimensional finite element model of a human lumbar annulus fibrosus. Clin Biomech.

[31] Polikeit A, Ferguson SJ, Nolte LP, Orr TE (2003). Factors influencing stresses in the lumbar spine after the insertion of intervertebral cages: finite element analysis. Eur Spine J.

[32] Carter DR, Hayes WC (1977). The compressive behavior of bone as a two-phase porous structure. J Bone Joint Surg Am.

[33] Shim CS, Park SW, Lee SH, Lim TJ, Chun K, Kim DH (2008). Biomechanical evaluation of an interspinous stabilizing device, Locker. Spine.

[34] Goto K, Tajima N, Chosa E, Totoribe K, Kubo S, Kuroki H, Arai T (2003). Effects of lumbar spinal fusion on the other lumbar intervertebral levels (three-dimensional finite element analysis). J Orthop Sci.

[35] Goel VK, Monroe BT, Gilbertson LG, Brinckmann P (1995). Interlaminar shear stresses and laminae separation in a disc. Finite element analysis of the L3-L4 motion segment subjected to axial compressive loads. Spine.

[36] Kim MC, Chung HT, Cho JL, Kim DJ, Chung NS (2013). Subsidence of polyetheretherketone cage after minimally invasive transforaminal lumbar interbody fusion. J Spinal Disord Tech.

[37] Schimmel JJ, Poeschmann MS, Horsting PP, Schonfeld DH, van Limbeek J, Pavlov PW. PEEK cages in lumbar fusion: Mid-term clinical outcome and radiological fusion. Clinical Spine Surgery. 2016; 29(5):E252-258. 10.1097/BSD.0b013e31826eaf74 27196005

[38] Talia AJ, Wong ML, Lau HC, Kaye AH (2015). Outcomes of extended transforaminal lumbar interbody fusion for lumbar spondylosis. J Clin Neurosci.

[39] Fogel GR, Turner AW, Dooley ZA, Cornwall GB (2014). Biomechanical stability of lateral interbody implants and supplemental fixation in a cadaveric degenerative spondylolisthesis model. Spine.

[40] Nayak AN, Gutierrez S, Billys JB, Santoni BG, Castellvi AE (2013). Biomechanics of lateral plate and pedicle screw constructs in lumbar spines instrumented at two levels with laterally placed interbody cages. Spine J.

[41] Goel VK, Lim TH, Gwon J, Chen JY, Winterbottom JM, Park JB, Weinstein JN, Ahn JY (1991). Effects of rigidity of an internal fixation device. A comprehensive biomechanical investigation. Spine.

[42] McAfee PC, Farey ID, Sutterlin CE, Gurr KR, Warden KE, Cunningham BW (1991). The effect of spinal implant rigidity on vertebral bone density. A canine model. Spine.

[43] Rohlmann A, Nabil Boustani H, Bergmann G, Zander T (2010). Effect of a pedicle-screw-based motion preservation system on lumbar spine biomechanics: a probabilistic finite element study with subsequent sensitivity analysis. J Biomech.

[44] Choi KC, Ryu KS, Lee SH, Kim YH, Lee SJ, Park CK (2013). Biomechanical comparison of anterior lumbar interbody fusion: stand-alone interbody cage versus interbody cage with pedicle screw fixation -- a finite element analysis. BMC Musculoskelet Disord.

[45] Johnson WM, Nichols TA, Jethwani D, Guiot BH (2007). In vitro biomechanical comparison of an anterior and anterolateral lumbar plate with posterior fixation following single-level anterior lumbar interbody fusion. J Neurosurg Spine.

[46] Xu H, Ju W, Xu N, Zhang X, Zhu X, Zhu L, Qian X, Wen F, Wu W, Jiang F (2013). Biomechanical comparison of transforaminal lumbar interbody fusion with 1 or 2 cages by finite-element analysis. Neurosurgery.

[47] Malham GM, Parker RM, Blecher CM, Seex KA. Assessment and classification of subsidence after lateral interbody fusion using serial computed tomography. J Neurosurg Spine. 2015; 23(5):589-97. 10.3171/2015.1.SPINE14566 26207320

